# “Ethics When You Least Expect It”: A Modular Approach to Short Course Data Ethics Instruction

**DOI:** 10.1007/s11948-020-00197-2

**Published:** 2020-02-17

**Authors:** Louise Bezuidenhout, Robert Quick, Hugh Shanahan

**Affiliations:** 1 Institute for Science, Innovation and Society, University of Oxford, Oxford, UK; 2 High Throughput Computing, Indiana University, Bloomington, IN USA; 3 Department of Computer Science, Royal Holloway, University of London, London, UK

**Keywords:** Data science, Data ethics, Open Science, CODATA, RDA

## Abstract

Data science skills are rapidly becoming a necessity in modern science. In response to this need, institutions and organizations around the world are developing research data science curricula to teach the programming and computational skills that are needed to build and maintain data infrastructures and maximize the use of available data. To date, however, few of these courses have included an explicit ethics component, and developing such components can be challenging. This paper describes a novel approach to teaching data ethics on short courses developed for the CODATA-RDA Schools for Research Data Science. The ethics content of these schools is centred on the concept of open and responsible (data) science citizenship that draws on virtue ethics to promote ethics of practice. Despite having little formal teaching time, this concept of citizenship is made central to the course by distributing ethics content across technical modules. Ethics instruction consists of a wide range of techniques, including stand-alone lectures, group discussions and mini-exercises linked to technical modules. This multi-level approach enables students to develop an understanding both of “responsible and open (data) science citizenship”, and of how such responsibilities are implemented in daily research practices within their home environment. This approach successfully locates ethics within daily data science practice, and allows students to see how small actions build into larger ethical concerns. This emphasises that ethics are not something “removed from daily research” or the remit of data generators/end users, but rather are a vital concern for all data scientists.

Modern life is exponentially generating an increasing amount of data (Hey and Trefethen 2003). Central to effectively navigating this “data deluge” are individuals skilled in the tools necessary to manage, curate and analyse data^1^ online. While such tasks have been historically within the remit of general data management and statistics, it is becoming apparent that the growing complexity of the data landscape requires wider familiarity with programming and computational skills. Fostering such skills within other disciplinary communities will strengthen data infrastructures and maximize the use of available data.

Building data science skill capacity within the research community broadly will also benefit contemporary research and evidence-based decision making. Indeed, modern research is increasingly becoming reliant on a range of data-related skills. These include research data management and curation, data platforms and infrastructures implementation, data analysis, statistics, visualisation and modelling techniques, software development, and annotation. There remains a shortage of individuals with these skills worldwide (Quick et al. 2017).

How best to teach these data science tools has been the topic of considerable discussion (Attwood et al. 2017). In particular, this is due to the inherent tension between two different conceptions of *who* is to be educated. Are the students disciplinary researchers with expertise in data analysis, or data analysts with expertise in disciplinary knowledge? In particular, formal teaching for the former is relatively new and the content of courses as well as pedagogical styles vary considerably.

In response to this global skill shortage, there has been an emergence of courses teaching data science skills to researchers outside of “traditional” data science disciplines such as computing and engineering. Many of these courses are not disciplinary-specific or run by individual institutions. Rather, there is growing support for short courses designed to teach introductory data science skills. Perhaps the most famous of these are Software/Data Carpentry short courses that employ a non-disciplinary, modular approach to teaching core computing skills.

If the content of current data science courses can be said to vary considerably, then the way/manner in which ethics instruction is provided is even more varied. Indeed, many technical courses teaching introductory coding, data management, manipulation and visualisation do not offer any specific ethics instruction. This is perhaps more surprising in light of the increasing concerns about research reproducibility (Kolker et al. 2014; Open Science Collaboration 2015; Resnik and Shamoo 2017), and the increasing scrutiny of data re-use (Ellison et al. 2011). The intensity of recent discussions around the use of personal Facebook data by Cambridge Analytica,^2^ for example, has highlighted these concerns. Discussions about the legitimate use of available data seem rarely out of the news. Interestingly—and perhaps unsurprisingly—within these discussions the responsibility of those harvesting and processing the data^3^ are heavily scrutinized. Indeed, there is an increasing expectation that those who have access to the data, and who design the programmes and platforms that facilitate its reuse, bear some responsibility towards the future applications.

Attempting to conceptualize how such a responsibility should be understood is, of course, no easy task. The distributed nature of data science, in terms of the origin of the data, the construction of the digital landscape and tools, and the analysis and development community, makes the application of traditional, individual-centric ethics discourse problematic. Indeed, traditional ways of framing responsibilities for individual conduct, such as the “FFP misconducts,” fabrication, falsification and plagiarism (National Academies of Sciences 2012), do not adequately encompass the responsibilities highlighted above. Apparently, the emerging models of scientific research that are, increasingly automated, data-driven and interdisciplinary, require a new type of ethics discourse.

Increasing the opportunities for data science training is vital for the advancement of modern research. However, with the acquisition of such tools comes responsibility. Students must recognize the ethical implications of being able to change, expand and diversify their research and use of data. How to integrate ethics into data science training remains an emerging topic of discussion. In addition to the challenges of overcrowded curricula, stand-alone short courses and interdisciplinary student groups, questions of content abound. This paper discusses the development of the SRDS ethics curriculum with the aim of stimulating discussion about innovative teaching and content-driven instruction.

## CODATA-RDA School for Research Data Science

The CODATA^4^-RDA^5^ Schools for Research Data Science (SRDS) were developed to offer data science training for early career researchers from low/middle-income countries (LMICs). Unlike many other courses, the SRDS curriculum was designed to not be “another bootcamp”. Rather, SRDS are two-week residential courses designed to build core data science skills, and to introduce open tools and resources for researchers. This curriculum, in the words of one of the founders (HS), is “broad and shallow” and introduces students to all the components necessary to build on for data science expertise.

Now in its fifth year, the SRDS network has hosted schools in Trieste, Italy (4 times), São Paulo, Brazil (2 times), and once each in San Jose, Costa Rica; Kigali, Rwanda; Addis Ababa, Ethiopia; Pretoria, South Africa and Brisbane, Australia (in an abridged format). In addition a number of new locations are planned in Africa and South America for the future. The schools are staffed by volunteer instructors and chaired by an international multidisciplinary committee. Each school also has a number of alumni who return as classroom helpers.

Building on the success of the Software/Data Carpentry model of instruction,^6^ the SRDS teaches key programming/data skills via short, intensive modules that iteratively build on one another. Technical topics covered during the SRDS include shell (command line), GitHub, R, SQL, Research Data Management, data visualisation, information security, machine learning, artificial neural networks and research computational infrastructures. Each module is highly interactive and involves both teachers and peer helpers to guide students through their practical learning.

All the schools have had a diverse student cohort. Attendees have been highly assorted in terms of disciplinary background, nationality, native language, age and gender. Similarly, the lecture pool is highly diverse, and comprised of volunteer instructors from a range of countries. Because of the diversity of nationalities within both the student and instructor groups, all instruction of the schools is in English.

From the outset, the SRDS chairing committee has been committed to the concepts of Open Science and Responsible Conduct of Research. Nonetheless, how best to transmit these values to the student cohorts was recognized to be a challenging undertaking. In particular, this was complicated by the integral characteristics of the schools, in that they had:

1. short timescales, intensive technical curricula
2. interdisciplinary student bodies
3. varying teaching staff from a network of summer schools.

Thus, the ethics curriculum should be accessible to instructors from multiple national and disciplinary backgrounds.

These characteristics challenged traditional forms of ethics instruction that utilize disciplinarily specific case studies or discussions on codes of conduct as teaching tools to facilitate student engagement (Miller 1988). Moreover, a small number of highly generalized, stand-alone lectures were deemed to be unhelpful in assisting students make connections between ethics and their daily research practices (Friedman and Kahn 1994, p. 67).

This paper discusses the decisions made by the SRDS to assist students in developing a practice-oriented understanding of data ethics. It is divided into three sections: identifying a central concept to orientate teaching, identifying content to teach, and finding ways to maximize student engagement with ethics throughout the 2-week course.

## Identifying a Central Concept to Orient Teaching

The students attending the SRDS did not necessarily have backgrounds in computing or engineering. Indeed, the majority of them were unlikely to self-identify as data scientists or computer scientists after graduating from the course. Because of this, it seemed limiting to link instruction too closely to guidelines for professional ethics originating from a specific discipline. Thus, while the excellent ethics literature and codes of ethics from key organizations such as the American Statistical Association and the Association for Computing Machinery^7^ informed the course development, no specific discipline was emphasized/highlighted in the course outline.

Instead, the course designers aimed to find a unifying concept that transcended disciplines and was accessible for students from different backgrounds and nationalities. The concept of *open and responsible (data) science citizenship* evolved naturally from the ethos of the SRDS: to educate responsible researchers who would be able to establish best practice within their home research environments. The program represents an Aristotelian view of citizenship as ethical obligations arising out of social living (Aristotle 1984). Being part of a community requires the acceptance of civic responsibilities and contribution to the overall public good. Enacting these responsibilities involves both the acknowledgement of civic duties and the fostering of specific virtues. In relation to the former, a good citizen is committed to following the rules, participating in civic activities and actively protecting/contributing to civic resources. In relation to the latter, the good citizen should be willing to foster the community by committing time and resources to necessary offices, charities and social causes.

The notion of “citizenship” is a useful way of highlighting the holistic nature of data ethics to the students. Just as one is a citizen of a country whether waking or sleeping, researchers are similarly bound and unable to pick and choose when to be a responsible citizen if one has agreed to the compact. This concept of “science citizenship” is presented to students using the following logic:

1. research is a community endeavour, and involves social actions such as resource sharing
2. the use of “community resources,” such as data, papers and so forth, is evidence of citizenship to the community and thus comes with civic responsibilities
3. these responsibilities include following community determined rules, including citation, licensing and so forth and contributing to civic resources, specifically data sharing
4. the responsibilities also include contributing to social good, by participating in civic service, for instance reviewing, and curating

Through the concept of citizenship it is possible to focus on/emphasize the reciprocal nature of responsibility. In other words, those benefitting from citizenship also have a responsibility to safeguard these benefits for others. The “citizenship” concept is a very important frame for excellence in data science practice as both an academic and social practice. This portrayal of an open and responsible science citizenship is also useful as it unites all students under a common cause and is not disciplinarily-dependent. The concept is presented to students as integral aspects of their identity as researchers that guide all aspects of their work. In the virtue ethics tradition, the concept of citizenship highlights how these responsibilities are indivisible from the identity of the researcher as a whole, and thus extends to all aspects of their work. This format supports a practice-based perspective on ethics that is contextually informed (MacIntyre 2007).

**Fig. 1:**
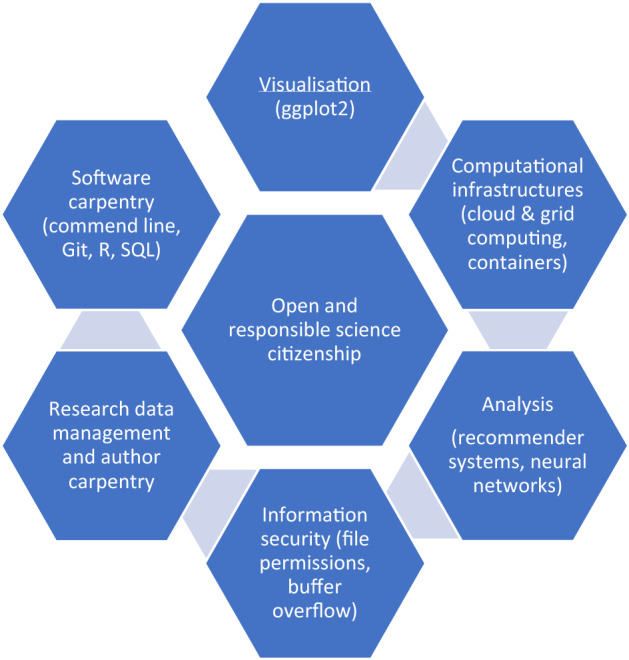
Schematic diagram of SRDS curriculum, highlighting centrality of open and responsible science citizenship

Figure 1 represents the SRDS curriculum as it is presented to the students. As can be seen, the concepts of openness and responsibility lie at the heart of the training, and inform all the technical content of the curriculum. Making this explicit, highlights two key issues. First, that ethical practice is integrated into daily research activities, and is not a stand-alone subject to be visited occasionally. Second, by learning data science skills, the students assume additional responsibilities to their communities. The students respond to community needs by being exemplars for best practice, by critically monitoring emergent digital infrastructures, or by developing ethical practice within their research communities.

This concept of science citizenship also includes a range of other topics that are specific to working with data. These include:

- Open Science
- infraethics and the design and deployment of Information and Communication Technologies (ICTs)
- appropriate data re-use (including ownership, consent, theft)
- credit and attribution for data re-use
- data management (including FAIR data^8^)
- Open Data (including open and reproducible authorship)
- responsibility to society and long-term beneficence resulting from the application of data science

## Identifying Content to Teach

After selecting open and responsible (data) science citizenship as the orienting concept, the course designers had to identify content to teach. The concept of science citizenship is extremely rich, and should be, of course, grounded in the individual ethics of responsible conduct of research (RCR). The field of RCR ethics addresses all aspects of personal responsible research conduct, ranging from misconduct (i.e., the “FFP” behaviours of fabrication, falsification and plagiarism) to treatment of colleagues, students and mentees (National Academies of Sciences 2012).

During the course the students needed to expand their understandings of responsibilities and duties in relation to data networks and infrastructures. As data science experts, the staff encourages them to recognize the role that they can play in scrutinizing the origins of data, the design of data infrastructures and the re-use of data, both in their own disciplines and more broadly. Indeed, there is an increasing expectation that those who have access to the data, and design the programmes and platforms that facilitate its reuse bear some responsibility regarding future applications. The topics to be addressed are briefly introduced below.

### An Ethics of Data Provenance

Discussions about data provenance have a long history in medical ethics and bioethics, particularly in relation to the use of personal/identifying data. These discussions hinge on the ethical obligations outlined in informed consent procedures and the responsibilities that data collectors and re-users have to respect them (Pearce and Smith 2011). Such concerns have come to the fore in the “omics era”, where disciplines such as genomics and proteomics are generating vast quantities of data containing individual identifiers (Kaye et al. 2009; Lunshof et al. 2008). Indeed, concerns of privacy, loss of autonomy or harm-via-identification are well-elaborated in these discussions. Together with less disciplinarily-specific discussions on scientific misconduct (National Academies of Sciences 2012), these topics have long dominated discussions on responsible data use. Nevertheless, the problems posed by the collection, analysis and re-use of large data sets in research, advertising and profiling are rapidly changing privacy discussions. Of particular concern is the possibility of re-identifying individuals from databases which are supposedly anonymised (Dwork 2008).

### An Ethics of Openness

Recent years have seen the rapid expansion of the Open Science movement. Open Data, as a subset of this movement, advocates for the unrestricted use of some data by all without restrictions from copyright, patents or other mechanisms of control (International Council for Science, InterAcademy Partnership, International Social Science Council, & World Academy of Science 2015). Commitments to openness are rapidly shaping discussions about data ownership sharing and re-use. Indeed, openness in data access and redistribution is rapidly becoming the de facto position for responsible research. The Open Data paradigm raises considerable ethical considerations with regards to the generation, recording, curation, processing, dissemination, sharing and use of data (Floridi and Taddeo 2016). Within this paradigm, data producers and users have considerable responsibilities to ensure that their data are accessible as prescribed by (the “FAIR” principles of data management. See Wilkinson et al. 2016), and reusable; that they adequately credit the use of other peoples’ data; and that they consider the potential future harms that could arise from disseminating the data they generate.

Ultimately, the sub-discipline of data ethics advocates for enhancing the use of data while respecting human rights and other values shaping open, pluralistic and tolerant information societies. In keeping with the Open Data movement, openness discussions advocate for open access to data but draws attention to the need to scrutinize the structures supporting openness of data availability and its reuse (International Council for Science, InterAcademy Partnership, International Social Science Council, & World Academy of Science 2015).

### An Ethics of Data Tools and Infrastructures

Data science is not solely about the re-use of data, but also the construction of data infrastructures and analysis tools. In a recent article Floridi and Taddeo (2016), sketched out this landscape in relation to “data ethics”. The authors differentiated this field from computer and information ethics, and posited that data ethics highlighted the need for scrutiny of the content and nature of computational operations. This, they suggested, raised several moral problems regarding algorithms, “including artificial intelligence, artificial agents, machine learning and robots … and corresponding practices (including responsible innovation, programming, hacking and professional codes)” (Floridi and Taddeo 2016, p. 1). The ethical concerns raised by algorithms focus on issues relating to the increasing complexity and autonomy of algorithms. Topics within this area are strongly linked to issues of artificial intelligence and machine learning, and highlight the crucial responsibilities and accountabilities of designers with regard to unintended or unforeseen consequences.

It is important to recognise that the potential moral problems identified by Floridi and Taddeo are highly scalable. Thus, decisions about what data sets to use, how to design algorithms, how to design databases and dissemination pathways—while seemingly innocuous to the individual practitioner—can lead to significant downstream ethical crises. This is well-illustrated by a number of now-famous case studies. For instance, the uncritical inclusion of cultural biases within algorithm design led to gender disparities within Google searches (Datta et al. 2015). Similarly, the uncritical use of databases and unreflective algorithm design led to the inappropriate distribution of targeted marketing of goods, and the violation of personal privacy by the Target supermarket group.^9^

Data ethics therefore draw attention to the need to examine “the interactions among hardware, software and data, rather than on the variety of digital technologies that enable them” (Floridi and Taddeo 2016, p. 1). This, of course, links strongly to the emergence of the Internet of Things (IoT), and highlights the need to examine the long-term implications of infrastructural and computational designs (infrastructure ethics, or “infraethics”). Data scientists therefore play a critical role in the creation of this emerging landscape, and need to be vigilant about identifying implicit biases, unintentional marginalization and future harms.

### An Ethics of Practice

Finally, the ethics of practice relate to the professional ethics of data users. Topics relating to this include the development of codes of conduct, policies and strategies that foster responsible innovation and sustainable progress. In this way, data ethics, as an ethics of design and implementation, relate to the emerging field of Responsible Research and Innovation (RRI). RRI champions the ethical development of technologies (Macnaghten et al. 2014; Owen et al. 2012; Stilgoe et al. 2013), particularly highlighting societal impact. The RRI movement offers a means of navigating the increasing complexity of links between science and society though movements such as citizen science, evidence-based policy making, and Open Data. Emphasizing the ethical nature of these links, RRI draws attention to key areas such as ethics, gender equality, governance, open access, public engagement and a commitment to education. Moreover, it highlights the need to consider these issues throughout the research cycle if responsible research is to be conducted.^10^ Such considerations are of even more importance when one considers the rapidly changing boundaries of public/private research, and of commercial/academic undertakings.

## Developing Teaching Formats That Maximize Student Engagement

The SRDS is taught in a 2-week block, and there is little formal teaching time allocated to ethics on the extremely full curriculum (3.5 h). Unlike more traditional ethics courses that take place over a semester or year with considerable student engagement time (Friedman and Kahn 1994), the course designers had to find ways to integrate open and responsible science citizenship in a short space of time. In particular, they had to find ways to integrate ethics into the curriculum so that it was integrated into the general flow of the course and was not a “stand alone” subject. Similarly, as the rest of the modules are highly practice-oriented, the staff had to ensure that the ethics instruction integrated into a highly technical curriculum in a way that nonetheless encourages internalisation and assimilation by the students.

Of course, the difficulties of integrating meaningful teaching into crowded curricula is a common occurrence for ethics education. Indeed, perceptions of “losing technical content to ethics instruction” is a common concern that ethics educators have to navigate (Miller 1988, p. 38). Similarly, high student numbers and limited time mean that there is often little space for more creative pedagogical tools, such as extensive group discussion, role playing, group work, projects or any of the other tools commonly promoted for engaged ethics pedagogy (Baker et al. 2013). Moreover, as there is often a lack of expertise in ethics teaching amongst computing faculty, there are concerns within teaching staff about taking on ethics teaching. In particular, potential educators are worried that lack of experience will lead to an imposition of moral codes rather than robust ethics discussions (Miller 1988).

As a result, lecturing ethics to science students often becomes a balancing act of content, depth, style and focus. Common responses to these balancing acts involve stand-alone ethics lectures detailing key ethical principles and/or case studies relating some ethical crisis. The limitations of this approach are evident. In particular, it is important to question how much the “stand-alone” style of ethics instruction enables students to internalize ethical norms and enact them in their daily practices (De Schrijver and Maesschalck 2013). It is often questioned whether an ethical “light touch” really leads to ethically competent researchers. Instead, detractors suggest that this educational approach educates solely for ethical awareness or compliance. Moreover, it is possible that the use of case studies—particularly those that do not closely reflect the working conditions and activities of the students—further hinder the process of internalization by making ethics appear as “something that happens to other people”. The challenge was therefore to find ways to address all these issues, and to weave ethics awareness throughout the curriculum.

As far as possible, the course designers wanted to avoid a stand-alone ethics lecture that provided a high-level introduction to ethical concepts without any contextualization. This approach to teaching, as discussed above, was felt to be unproductive and stopped students from making connections between ethics and their daily practices. The course designers were very sure that what is needed is a combination of broader ethical principles with contextual case studies enables students to see how the ethical principles translate into daily practice. It was also important that these discussions include all key areas of data ethics: provenance, design of infrastructures and practice.

The course designers determined that ethics need to be embedded at the core of the SRDS curriculum. Students need to see how ethics permeate all aspects of data science practice, from their use of programming tools to their authorship practices and research data management. In collaboration with both Sarah Jones, the research data management instructor, and Gail Clement, the open authorship instructor, the design team capitalized on the allocated teaching time within the curriculum to maximise the exposure that the students had to open and responsible science citizenship (Table 1).

**Table 1:** Course breakdown for teaching open and responsible (data) science citizenship

Subject	Topics covered	Number of hours
Research data management	Data management, data management plans, FAIR data, repositories	5 h + 4 h practical
Open authorship	Reproducible reporting, DOIs, data licensing, ORCIDs	4.5 h
Responsible conduct of research (RCR) and Open Science	Introduction to ethics, RCR, overview of open science, contextualizing openness and responsibility	3.5 h
Technical data skills	Ethics exercises linked to technical content (see Fig. 2)	Variable

FAIR data refers to the movement to develop standards to make data Findable, Accessible, Interoperable and Reusable. DOI refers to Digital Object Identifier

### Lecture 1: Introducing Key Concepts

In all the summer schools, the majority of students have no prior exposure to formal ethics instruction. The first lecture of each SRDS therefore, addresses the key concepts of open and responsible data science citizenship. This 1.5 h time slot consists of a lecture introducing key concepts such as Open Science/Data, Responsible Conduct of Research and the course designers’ concept of open and responsible data science citizenship. The lecture is followed up by a series of exercises during which students are asked to note issues they felt represented good and bad practice in relation to research data. Students first note their own perceptions, then discuss synergies in small groups. This is followed by a group discussion.

### Week 1: Lectures

During week 1 students are taught a range of technical modules, including shell, R, GitHub, and SQL. As will be discussed below, each of these modules involves some ethics instruction. Week 1 also includes modules on research data management and open authorship. These modules are strongly rooted in both Open Science and Responsible Conduct of Research. Furthermore, these modules introduce specific data ethics issues relating to the subject area. For example, research data management includes discussions on FAIR data (Wilkinson et al. 2016), while open authorship includes discussions on predatory journals, author processing charges, and channels of data dissemination.

### Lecture 2: Contextualizing Ethics

Week 1 ends with a 2-h ethics lecture. This lecture has two key objectives: to enable and prompt students to begin to think about how to implement open and responsible science citizenship within their own research context, and to think about the broader responsibilities associated with data science expertise.

Persuading students to problematize how they would implement open and responsible science citizenship within their own research institution is linked to the underlying virtue ethics tradition informing the SRDS curriculum. As the curriculum advocates for practice-based ethics, it is vital that students start making connections between the ethics instruction they are receiving and their daily research practices. Moreover, as the students of the SRDS are from LMICs, it is likely that many of them will experience challenging circumstances in their home research environments. Enabling students to discuss potential challenges to openness and responsibility is thus an important way of normalizing future problems, of highlighting potential solutions, and of ensuring that students feel comfortable raising these challenges with their peers and future collaborators. This ensures the longevity of the SRDS instruction and avoids students becoming disheartened and disengaged.

The discussions on challenges and solutions makes use of the grid presented in “Appendix 1” This is given as a handout to students, who are encouraged to fill it in during and after their discussions. As is evident from the design, the object of the grid is to get students to think about their challenges and practices through the research life cycle. The second column of “Appendix 1” lists some of the tools that are introduced during the SRDS, column 3 highlights some of the ethical issues that were discussed, and columns 4 and 5 require the students to fill in issues relating to their own context. Columns 2 and 3 are intentionally incomplete, requiring the student groups adapt them as they see fit.

Once the students complete their grids, the class engages in a group discussion about the challenges of implementing open and responsible data science citizenship within one’s home institution, and problematizes ways in which these challenges can be overcome. Common issues to be discussed include institutional cultures such as, promotion criteria, incentivization, cultural specificities; institutional support such as facilities, resources, institutional cultures; resources, such as time, money, infrastructures; copyright and ownership, and general concerns such as being scooped and not having time for research.

By getting students to talk through the problems and possible solutions the staff hope to demystified some of the misconceptions about Open Data: that it should be easy, that other people do not have problems, that if someone cannot get it right it is their own fault. The staff encourages students to see that their peers, and even the instructors, experience the same problems and that the most effective way of dealing with them is to ask for help. Students need to identify how the multifarious tools that they have learned during the SRDS can to proactive problem-solving actions.

At the end of the class the staff encourages students to form support networks that they can tap into once they return home. In particular, students who would likely experience similar problems at their home institutions are encouraged to connect so as to share best-practice experiences and ideas. Having this support is something the staff views as essential for stable and persistent ethics among outgoing students.

The second half of this session involves a more formal lecture introducing some of the broader topics of data ethics, such as infraethics and algorithmic biases. This sets the scene for the ethics exercises relating to the modules offered in the second week such as data visualization, information security, recommender systems, machine learning, and research computational infrastructures.

### Modular Ethics Exercises

**Fig. 2:**
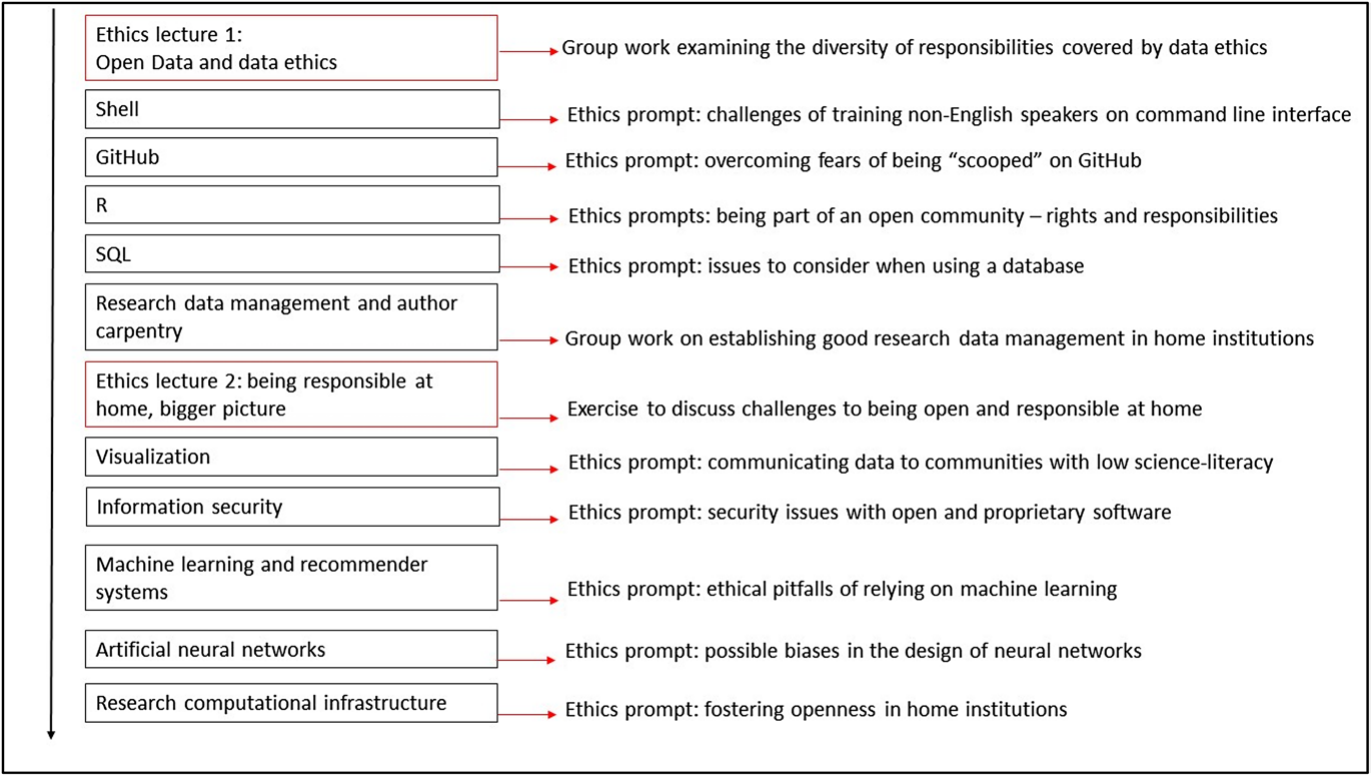
Structure of the SRDS demonstrating distribution of ethics prompts

As mentioned above, the SRDS curriculum is modular, and students learn key data science tools in discrete work packages. This approach follows the Carpentries format, which is modular and incremental (Teal et al. 2015). In order to ensure that the ethics content from the formal ethics lectures is linked to the technical content, the course designers created small 15-min “ethics prompts” to accompany each module (see Fig. 2). The ethics prompts are administered via a range of different modalities, including writing answers on post-it notes, live voting and mind-mapping. Students are expected to complete an ethics-related question and participate in a short discussion at the end of each module (see “Appendix 2”). These ethics prompts are specifically related to the content of the module completed, while linked to the broader ethical issues introduced in the lectures.

The prompts are intended to link the concept of *open and responsible science citizenship* to the data tools being taught. Engaging students in a short amount of ethical reflection that relates to the tool they have just learned is a good way of highlighting/noting ethical issues, responsibilities and considerations that are part of daily data science practice. They also provide the opportunity to extend the ethics discussions to some topics that could not be addressed in the formal instruction. The responses to each prompt are collated by the organizers, and a summary of the class participation for each prompt are displayed on boards in the communal area for the duration of the course. Students often visit these boards during the break times, showing a good level of follow-up on the exercises.

## A Reproducible Curriculum for Open and Responsible Science Citizenship

By integrating ethics content into technical lectures, making use of short discussion questions, and continually revisiting the central concept of citizenship, the SRDS is able to provide the students with a broad overview of data ethics. While this instruction is, of course, by no means comprehensive, it nonetheless significantly expands students’ ethics engagement beyond that possible in 3.5 h of teaching. The distribution of the content across the different learning modalities is displayed in Table 2.

**Table 2:** Schematic representation of distribution of content

Element of science citizenship	Student engagement activities
Ethics and RCR	Lecture 1, Lecture 2
Openness and Open Science	Author carpentry, RDM
Data provenance	RDM, SQL EP,
Data tools and infrastructure	Lecture 2, GitHub EP, Security EP, Recommender EP, Neural Networks EP, Computational Infrastructures EP
Data practices	Shell EP, R EP, Data Visualization EP

The full design of the curriculum can be seen on GitHub and is free to download.^11^ A key part of the SRDS vision is to provide an open curriculum that can be reproduced in summer schools around the world. As this curriculum has been taught to multi-disciplinary and -national student groups around the world, it is anticipated that the curriculum can be easily adapted for different contexts. Moreover, the curriculum has been taught both by ethics educators as well as by those from other disciplines. This suggests that the content and format support teachers who may be apprehensive about teaching outside of their field of expertise. In response to this need the course designers purposively devised the module using an iterative approach that involves considerable group work and discussion. This takes the pressure off instructors who might be uncomfortable with long, theory-focused lectures. By basing ethics education in practice, the course designers also hope to enable potential instructors to immediately see how, where and why open and responsible (data) science citizenship fits into the SRDS design.

While the SRDS are only two weeks, the course designers hope that the multi-modal form of ethics engagement sparks the interest of students and enables them to see both the “microethical” issues of professional responsibility and the “macroethical” issues of technology (Herkert 2005). By being purposively disciplinarily non-specific, the hope is that students will also recognize their membership to a broader data science community, as well as to their own discipline and national communities.

Being disciplinarily non-specific does, however, come with problems, as it makes the use of existing resources in the form of case studies and codes of ethics (Herkert 2010) difficult. Nonetheless, the creation of the module-linked ethics prompts may serve as micro-case studies, and stimulate discussion amongst students. Indeed, the use of the ethics prompts instead of full case studies may also be beneficial for transporting the curriculum around the world, where many future teachers have little experience teaching ethics or utilizing discussion-based pedagogical tools (Martin 1997).

### Student Responses

The strength of the approach is perhaps best summed up in the words of one of the 2016 students, Marcela Córdoba: “Once we [her colleagues at her home university] started connecting the need for more resources to practice Open Science, i.e. institutional data repositories and open data policies, funding to pay for publication in open journals, support for learning about reproducible tools, etc., with the need for the skills of Data Science in other departments (since we have students from other departments constantly consulting our department about data analysis), we felt that something like the CODATA-RDA school was needed in Costa Rica…. CODATA-RDA schools changed my career, making me a more responsible researcher but also an Open Science ambassador for the Central American area. I now aspire to be a young researcher that can teach Open and Data Science principles through my job at the University of Costa Rica and through the CODATA-RDA Schools, as well as also serve as a mentor for other people that want to learn how to practice Open Science.”^12^

All aspects of the data ethics module have received positive reviews from the students attending the SRDSs. In particular, the final discussion on contextualizing open and responsible (data) science citizenship always elicits considerable discussion that students find useful. Since the first SRDS the authors have also seen a number of students engage in Open Science activities (seminar organizing, blogging and research projects) in their home environments. We believe that this suggests that this method of teaching ethics offers a valuable and long-lasting way of introducing ethics within an extremely time-constrained curriculum.

More research is needed to assess the long-term internalization of data ethics by the student body, or to compare this approach to more traditional teaching methods. Nonetheless, a case can be made for the further expansion of this method in teaching data ethics. First, considerable evidence suggests that the repetition and reiteration of ethics issues strengthens the likelihood of internalization and assimilation by the student. Second, associating ethics with practical skills used in daily research prevents ethics from being “something that is done once in the course of a project”. Finally, and perhaps most importantly, this approach demonstrates to students that ethical issues permeate data sciences regardless of discipline or the origin of the data set.

### Suggestions for the Future

The initial success of this approach to data ethics education is encouraging. Not only did the students enjoy the varied format of the instruction, but the format offered some key additional benefits. On a practical level, the combination of ethics lectures with both group discussions and short, targeted ethics activities increased the exposure of students to ethical concerns. On a pedagogical level, it is possible that tethering the ethics content to key tools used in daily research sensitized students to the ethical content of their daily research practices. Nonetheless, as mentioned above, more needs to be done to evaluate this practice-oriented approach to teaching data ethics. In particular, the development of monitoring and comparative evaluation strategies between different modules using this approach would be very helpful. Moreover, the creation and population of a repository of content-related ethical prompts and suggestions for delivery would benefit the entire community of data ethics instructors.

Whatever comes next, it is time to start thinking about how best to teach data ethics effectively and efficiently. It is unlikely that the space given to ethics in data science curricula will increase significantly, so it is up to the data ethics community to find creative ways around existing limitations. It is hoped that this paper offers a start to these conversations.

## Appendix 1: Tables to Contextualise Challenges to Open and Responsible Science Citizenship in Home Environments Using Research Lifecycle and RCR Frameworks

### Research Lifecycle

**Table:**

Research activity	Tools and instruments	Key ethical considerations	Key challenges in your research environment	How Can I Get Assistance?
Create	R, GitHub (versioning), data management plans, research data mamangement,,, FAIR data checklists, ethical approval, EOSC, FOSTER, OpenAIRE	Is my research ethical and responsible? Have I considered all types of data that I am producing? Have I thought about how to implement FAIR and openness?		
Document	GitHub Research Data management FAIR data checklists OMERO	Is my metadata sufficient to allow for scrutiny and re-use?		
Use	Analysis, GitHub, R, specific analysis tools	Am I using open analysis tools? Am I complying with the ethical requirements for secondary data use?		
Store	Storage and backup options Dropbox	Are my data properly curated and annotated for re-use? What are the implications of 3^rd^ party, commercial storage?		
Share	Licensing—Creative Commons Re3data, DOIs, EOSC, FOSTER, OpenAIRE	Am I using open, sustainable and responsible pathways to sharing? Could my data be misused for negative purposes? How will I control for this?		
Preserve	Archiving	Is my data guaranteed to have long-term preservation?		

### Responsible Conduct of Research

**Table:**

Research activity	Tools and Instruments	Key ethical considerations	Key challenges in your research environment	How can I get assistance?
Research misconduct	RCR guidelines, Open Data guidelines, Institutional policies	Fabrication, falsification and plagiarism Research causing harms Lack of attribution/respect of licensing		
Conflicts of interest and commitment	RCR guidelines	Biases in research caused by undisclosed conflicts		
Collaborative research	Journal guidelines, Memoranda of Understandings Licensing	Poor attribution of credit Scooping, theft, loss of control of data		
Authorship and publications	AuthorAid ORCID	Attribution of credit Open Access		
Peer review	Publons	Theft of ideas Uncollegial behaviour (bullying, unfair review, etc.)		
Mentorship and trainee relationships		Appropriation of student’s research Failure in duty to teach Failure in duty to care Teaching in an appropriate fashion		

## Appendix 2: List of Ethics Prompts

**Table:**

Module	Question	Response
GitHub	Content on GitHub can only be made private with a subscription fee. Does the idea of having unpublished work freely open and accessible to anyone bother you? Yes/no	It’s ok to be concerned. Thinking carefully about where and how your share is responsible But it is important to recognize that all content online is “published” in terms of legal and ethical standards—“published” = making public GitHub and other sharing sites offer the ability to attach legal licenses that require attribution, i.e., Creative commons Some sharing sites offer the opportunity to add disclaimers for downstream use of data Registering outputs as DOIs provides a unique identifier for citing your work Not all data/projects are appropriate for GitHub, i.e., Data with ethical requirements. Be sure you’ve thought through how your work can be used downstream
R	R is an example of Free and Open Source Software. It is a community-originated product, and users do not have access to technical support in the same way they would have as license holders of proprietary software. Users thus rely on community forums and peer support for assistance when they run into problems. These community forums rely entirely on the time of volunteer users As a future R user, how important do you think it is to dedicate time to helping other users on community forums? 1. Not really, there are enough people helping 2. I’d like to, but I don’t have the time 3. I’d like to, but I don’t feel that I have the expertise 4. I will try from time to time 5. I will contribute regularly	Contributing to communities online: Access to Open Science resources is both a right and a responsibility Open Science movements are only as strong as their members Being open is a kind of gift economy—we receive gifts/opportunities but must be willing to pay back without expectation of reward Engaging with a community can lead to unexpected benefits—i.e., Learning, collaborations, visibility/prestige, friendships Following community activities—even if you’re not ready to contribute—can be very useful as you will: Get used to how the community operates Identify leaders to follow Learn from discussions Become part of a global community that links you across the globe
SQL	SQL enables communication with these databases, which makes it a powerful tool in research. Many of the databases/datasets that you will be using will be open. This means that they are available for re-use, but also means that you have a responsibility regarding how you re-use them. Please select all actions you should always take before using the database Nothing, if the data are open it is free to be re-used Nothing before I use the data, but I will give credit to the data producer after I use it I must check the metadata for any information about the ethical commitments made by the data producer I must contact the original data producer to tell them about my research I must alert my department so that they can register IP I must check whether the data are licensed by a Creative Commons license I must check how the methods by which the original data were produced to ensure that they were responsibly produced I must check that the data have been reused in other published papers I must email the database curator my data management plan	Always I must check the metadata for any information about the ethical commitments made by the data producer I must check whether the data are licensed by a Creative Commons license I must check how the methods by which the original data were produced to ensure that they were responsibly produced Good, but not necessary I must contact the original data producers to tell them about my research I must check that the data have been reused in other published papers I must email the database curator my data management plan Never Nothing, if the data are open it is free to be re-used Nothing before I use the data, but I will give credit to the data producer after I use it I must alert my department so that they can register IP Using Open Data/bases is a privilege and a responsibility You can show your respect for the data you use by being as open and transparent as possible However, before using any data—open or not—you must always Check for the ethical commitments attached to the data—check the metadata, but if in doubt email the original producer Check licensing—even Open Data may have restrictions on use. Check the CC website for descriptions on the different licenses Check the methods by which the data were produced—was it responsible research practice? Is it robust and reproducible research
Data visualisation	Exercise adapted from O’Brien, 2017 Full thesis available here: https://repository.asu.edu/attachments/194100/content/OBrien_asu_0010N_17523.pdf	Data visualizations are used to communicate information about important social issues to large audiences Ethical problems in data visualizations can be intentional or unintentional Visualization may use deceptive techniques that have the potential to alter the audience’s understanding of the information being presented Common deceptive data visualization techniques including message exaggeration/understatement and message reversal (i.e. Flipping or inverting axis of chart) Data visualizations carry the same ethical importance as other forms of communication Similar to journalists, technical communicators must follow a set code of ethics. According to the Society for Technical Communication (STC), “as technical communicators, we observe the following ethical principles in our professional activities” listing legality, honesty, confidentiality, quality, fairness, and professionalism as the main ethical categories for technical communicators www.stc.org/about-stc/ethical-principles/ (accessed 7 February 2020)
Information security	Q1: Is Open Software likely to be more or less secure than proprietary software? Q2: You need to encrypt sensitive data for an international research group. The government in the country where you live mandates a certain encryption technology and is widely suspected of leaving “backdoors” (i.e., ways to access the data without knowing the encryption key). How do you respond?	A1: Generally, more secure if there are active contributors reviewing the code and resolving security issues. However, it is also easier for attackers to understand the code and look for security holes A2: There are multiple aspects to it. In some cases you may have no choice but to follow the law, in others it may make sense for the data to be stored in a different country since there are multiple countries in the collaboration. Even if you trust your government, encryption backdoors can also be exploited by attackers
Recommender systems	Minneapolis, 2012 Target is a large retail firm in the USA that uses data analytics and recommender systems to tailor coupons to their customers. In Minneapolis in 2012 a customer approached the manager “My daughter got this in the mail!” he said. “She’s still in high school, and you’re sending her coupons for baby clothes and cribs? Are you trying to encourage her to get pregnant?” The manager didn’t have any idea what the man was talking about. He looked at the mailer. Sure enough, it was addressed to the man’s daughter and contained advertisements for maternity, nursery furniture and pictures of smiling infants. The manager apologized and then called a few days later to apologize again On the phone, though, the father was somewhat abashed. “I had a talk with my daughter,” he said. “It turns out there’s been some activities in my house I haven’t been completely aware of. She’s due in August. I owe you an apology.” The selection of coupons that the daughter received were based on the data collected from her store loyalty card. Changes in purchasing behaviour were linked to certain likely outcomes, leading to the receipt of pregnancy-related coupons. Select all the statements you agree with: 1. I think that targeted marketing is unproblematic 2. If the daughter accepted a store loyalty card, she should have read the terms and conditions and accept the consequences 3. I think that there should have been some filters built into avoid targeting under-18s 4. I think that it is not right that Target employees are placed in situations where they cannot explain their company’s marketing decisions 5. I think that it is not right that Target has the ability to impose on the privacy of the daughter 6. I think that directed marketing can only be used for certain medical conditions (like pregnancy) but not others (like sexually transmitted diseases) 7. I think that directed marketing can cause harm as it should not be assumed that all women respond to their pregnancy in the same way	Recommender systems should not be thought of as neutral The choice of datasets to utilize, the links made and the responses prompted all reflect specific cultural values and assumptions While including these values are not necessarily unethical in their own right, it is important to recognize that they can have unintended consequences It is important for developers of recommender systems to be aware of the values that they introduce into the system, and their implications for the broader society
Artificial neural networks	Neural networks (and other machine learning techniques) can be trained to identify different types of”events” from data. One application that is currently very topical is to identify terrorists (from web browser history, travel, purchases, who they communicate with, etc.). The use of such applications by the state raises concerns about human rights and excessive surveillance. Other concerns, of course, relate to the mis-identification of individuals, as can be anticipated a small percentage of the time Suppose such a technique identifies an individual as having a high probability of committing a terrorist act in the near future. Maybe 95%—and that’s an honest number, not the prosecutor’s fallacy. But he/she has not broken any law (yet) and the neural net just says “this is typical terrorist behaviour”: it does not give any reason This, of course, leads to a number of different possibilities. For instance, some governments would curtail their liberty, even though they are innocent. Others might choose not to intervene, even though people may die. If you take two patterns which are identical except for the religion, or ethnicity, of the individual, your neural net will probably give very different answers. What answer best reflects your position: 1. Distinguishing individuals by religion or ethnicity perpetuates stereotypes and should not be used to distinguish individuals 2. Using all resources available is justified to save lives, thus the use of neural networks is justified 3. It is unacceptable to impose on individual freedom through pre-emptive interventions based on neural networks 4. The use of neural networks should only be used by the government on its own citizens. Being part of the online environment is not an invitation for foreign powers to use information about non-citizen individuals	The use of machine learning techniques for civil governance is very controversial and opinions are very divided as to whether they are just A problematic aspect of these techniques is that the general public has little likelihood of understanding how these systems are set up The users are also unlikely to share details for fear that the systems can be hacked, gamed or appropriated Many decisions made by users of these systems go unchallenged Data scientists are in a good position to monitor the development and deployment of such systems due to their ability to engage with the technical aspects of these systems
Research computational infrastructures	The Association for Computing Machinery has the following code of ethics: https://www.acm.org/code-of-ethics (accessed 7 February 2020) Exercise: read the code at Pay special attention to section 3.7 that deals with computational infrastructures	Recognize and take special care of systems that become integrated into the infrastructure of society Even the simplest computer systems have the potential to impact all aspects of society when integrated with everyday activities such as commerce, travel, government, healthcare, and education. When organizations and groups develop systems that become an important part of the infrastructure of society, their leaders have an added responsibility to be good stewards of these systems Part of that stewardship requires establishing policies for fair system access, including for those who may have been excluded. That stewardship also requires that computing professionals monitor the level of integration of their systems into the infrastructure of society. As the level of adoption changes, the ethical responsibilities of the organization or group are likely to change as well. Continual monitoring of how society is using a system will allow the organization or group to remain consistent with their ethical obligations outlined in the Code. When appropriate standards of care do not exist, computing professionals have a duty to ensure they are developed

## Footnotes

1 Implicitly the assumption being made is that data are those elements that can be stored electronically (at their most basic level, as streams of binary numbers) and hence accessible over the Internet. As noted by Christine Borgman, no one definition of data exists, especially in a research-based context, but would have a wider definition than the above, for example including physical artefacts (Borgman 2015). However, the expansion encountered above has been in the more specific class, with which disciplines such as computer science would be familiar, and hence this paper will work in this context.

2 http://www.bbc.co.uk/news/uk-43474760 (accessed 21/03/2018).

3 http://www.bbc.co.uk/news/uk-43480978 (accessed 21/03/2018).

4 Codata is the Committee on Data of the International Science Council.

5 RDA is the Research Data Alliance.

6 https://software-carpentry.org/ (accessed 22/03/2018).

7 https://www.amstat.org/ASA/Your-Career/Ethical-Guidelines-for-Statistical-Practice.aspx https://www.acm.org/code-of-ethics (accessed 15/07/2019).

8 “FAIR” are a set of guiding principles to make data Findable, Accessible, Interoperable, and Reusable.

9 http://www.workplaceethicsadvice.com/2012/02/target-sends-coupons-to-pregnant-girl-and-unawares-dad-explode.html (accessed 22/03/2018).

10 See rri-tools.eu (accessed 22/03/2018).

11 https://github.com/CODATA-RDA-DataScienceSchools/Materials.

12 My journey towards Open Science: How CODATA-RDA Summer Schools changed my career. January 27, 2018. https://researchdata.springernature.com/users/81847-marcela-alfaro-cordoba/posts/29656-my-journey-towards-open-science (accessed 21 March 2018).
